# Early dysfunctions of fronto-parietal praxis networks in Parkinson’s disease

**DOI:** 10.1007/s11682-016-9532-7

**Published:** 2016-03-02

**Authors:** Eva Matt, Thomas Foki, Florian Fischmeister, Walter Pirker, Dietrich Haubenberger, Jakob Rath, Johann Lehrner, Eduard Auff, Roland Beisteiner

**Affiliations:** 1 Department of Neurology, Medical University of Vienna, Währinger Gürtel 18-20, A-1090 Vienna, Austria; 2 MR Centre of Excellence, Medical University of Vienna, Währinger Gürtel 18-20, A-1090 Vienna, Austria; 3 NINDS Intramural Research Program, National Institutes of Health, 9000 Rockville Pike, Bethesda, MD 20892 USA; 4 Department of Neurology, MR Centre of Excellence, Medical University of Vienna, Währinger Gürtel 18-20, A-1090 Vienna, Austria

**Keywords:** Parkinson’s disease, fMRI, Apraxia, Praxis network, Dopaminergic therapy

## Abstract

In Parkinson’s disease (PD) the prevalence of apraxia increases with disease severity implying that patients in early stages may already have subclinical deficits. The aim of this exploratory fMRI study was to investigate if subclinical aberrations of the praxis network are already present in patients with early PD. In previous functional imaging literature only data on basal motor functions in PD exists. Thirteen patients with mild parkinsonian symptoms and without clinically diagnosed apraxia and 14 healthy controls entered this study. During fMRI participants performed a pantomime task in which they imitated the use of visually presented objects. Patients were measured ON and OFF dopaminergic therapy to evaluate a potential medication effect on praxis abilities and related brain functions. Although none of the patients was apraxic according to De Renzi ideomotor scores (range 62–72), patients OFF showed significantly lower praxis scores than controls. Patients exhibited significant hyperactivation in left fronto-parietal core areas of the praxis network. Frontal activations were clearly dominant in patients and were correlated with lower individual praxis scores. We conclude that early PD patients already show characteristic signs of praxis network dysfunctions and rely on specific hyperactivations to avoid clinically evident apraxic symptoms. Subclinical apraxic deficits were shown to correlate with an activation shift from left parietal to left frontal areas implying a prospective individual imaging marker for incipient apraxia.

## Background

Apraxia is a higher-order motor disorder resulting from brain disease affecting the performance of learned, skilled movements (Leiguarda and Marsden 2000). Apraxic patients frequently exhibit deficits in pantomiming and actual use of objects or tools and in imitating gestures (Geschwind and Damasio 1985). Praxis deficits have been shown to have adverse effects on activities of daily living, e.g. mealtime behavior or body hygiene (Foundas et al. 1995; Hanna-Pladdy et al. 2003; Vanbellingen et al. 2012) and to hinder the rehabilitation progress (Dovern et al. 2012; Bieńkiewicz et al. 2014). Importantly, these apraxic symptoms cannot be explained by basal motor or sensory deficits and are thus considered as impairments of central motor planning (Rothi and Heilman 1997). Although basal motor functions in PD have already been investigated with functional imaging, no fMRI investigation of praxis functions in PD or other clinical populations exists so far.

### Neuronal basis of praxis (dys)functions

Structural lesion studies in stroke or tumor patients and functional imaging studies in healthy subjects suggest that praxis functions are organized in a widespread left-hemispheric cortical network of parietal, frontal, and temporal areas (Niessen et al. 2014). Praxis abilities have been investigated using a broad spectrum of tasks addressing perception, imagination, imitation, and pantomiming of transitive (object related) and intransitive (expressive or communicative) gestures as well as the actual use of objects or tools (Bieńkiewicz et al. 2014).

A recent model (Buxbaum and Kalénine 2010; Binkofski and Buxbaum 2013) proposes that gesture production based on visual input recruits two distinct pathways within the dorsal visual processing stream (Ungerleider and Mishkin 1982; Milner and Goodale 1995). According to this model a bilateral dorso-dorsal stream incorporating superior occipital areas, the intraparietal sulcus, the superior parietal lobe, and the dorsal premotor cortex subserves online monitoring of actions directed at visible stimuli on the basis of their structural properties such as reaching towards an object or grasping (Binkofski and Buxbaum 2013). Indeed, lesions in the dorso-dorsal stream are associated with optic ataxia characterized by misreaching (Perenin and Vighetto 1988; Karnath and Perenin 2005), deficits in grasping (Tunik et al. 2005), but also with imitation deficits (Hoeren et al. 2014). The left-hemispheric ventro-dorsal stream running through medial superior temporal areas, the inferior parietal lobe, and the ventral premotor cortex is thought to contain long-term representations of skilled object-related actions (Heilman et al. 1982) including the ability to make inferences about the function of an object from its structure (Binkofski and Buxbaum 2013). Accordingly, lesions in the ventro-dorsal stream were found to produce deficits in pantomime or real use of objects, especially when the inferior parietal lobe is affected (Heilman et al. 1982; Buxbaum et al. 2007; Goldenberg and Spatt 2009; Randerath et al. 2010; Hoeren et al. 2014; Weiss et al. 2014; Buxbaum et al. 2014; Watson and Buxbaum 2015). In line with findings from lesion studies, functional imaging studies demonstrated the involvement of left parietal areas in different praxis task such as planning and execution of pantomiming or real use of objects (Choi et al. 2001; Ohgami et al. 2004; Fridman et al. 2006; Imazu et al. 2007; Króliczak and Frey 2009; Vingerhoets et al. 2011; Brandi et al. 2014; Mäki-Marttunen et al. 2014; Vry et al. 2015).

Frontal regions associated with apraxia comprise left inferior and middle frontal gyri (Buxbaum et al. 2003; Goldenberg et al. 2007; Hermsdörfer et al. 2013, Manuel et al. 2013; Buxbaum et al. 2014) as well as primary motor and pre-motor areas (Goldenberg et al. 2007; Huey et al. 2009; Weiss et al. 2014; Watson and Buxbaum 2015). Consistent with these findings, functional imaging studies reported frontal areas to be commonly co-activated with parietal regions during praxis tasks (Choi et al. 2001; Ohgami et al. 2004; Fridman et al. 2006; Króliczak and Frey 2009; Mäki-Marttunen et al. 2014; Vry et al. 2015) that is likely to be mediated via fiber tracts connecting these regions (Vry et al. 2015). Indeed, lesions or degeneration of fronto-parietal connecting fiber tracts were found to impair praxis abilities (Kertesz and Ferro 1984; Zadikoff and Lang 2005; Borroni et al. 2008; Manuel et al. 2013). Moreover, left temporal regions were also found to be involved in praxis as reported from functional imaging studies (Choi et al. 2001; Johnson-Frey et al. 2005; Vingerhoets et al. 2011; Mäki-Marttunen et al. 2014; Lausberg et al. 2015; Vry et al. 2015) or lesion studies (Buxbaum et al. 2014; Watson and Buxbaum 2015). Additional, subcortical lesions can lead to apraxia as reported for the thalamus (Pramstaller and Marsden 1996; Buxbaum et al. 2014) and the basal ganglia (Pramstaller and Marsden 1996; Hanna-Pladdy et al. 2001; Huey et al. 2009).

### Apraxia in Parkinson’s disease

Apraxia occurs in a large variety of neurological diseases and plays a major role in movement disorders including Parkinson’s disease (PD), Huntington’s disease (Hödl et al. 2008), primary cervical dystonia (Hoffland et al. 2011), and in corticobasal degeneration (Zadikoff and Lang 2005). In movement disorders a valid assessment is difficult since praxis deficits are superimposed on elementary motor dysfunctions like bradykinesia (Zadikoff and Lang 2005; Vanbellingen et al. 2012). In PD, 17–64 % of the patients exhibit apraxic symptoms (Grossman et al. 1991; Leiguarda et al. 1997; Vanbellingen et al. 2012). This variability in prevalence figures can most likely be attributed to variable apraxia assessments and patients investigated at different disease stages. Indeed, Vanbellingen et al. (2012) reported that the frequency of apraxia increases with disease severity from 0 % in Hoehn and Yahr stage 1 up to nearly 40 % in stage 4. Apraxia in PD patients is mainly characterized by spatial errors and difficulties in imitating hand and finger posture, while gesture comprehension, recognition, and discrimination are preserved (Leiguarda et al. 1997). The latter study also report that apraxic deficits do not improve with dopaminergic treatment. Importantly, apraxia scores in PD are not correlated with motor disabilities (Goldenberg et al. 1986; Grossman et al. 1991; Leiguarda et al. 1997; Vanbellingen et al. 2012).

### Functional imaging of basal motor and praxis functions

Up to now, no functional imaging study has addressed praxis (dys)functions in PD. In patients with apraxia due to corticobasal degeneration or stroke a few small studies reported metabolic and electrophysiological abnormalities. At rest, apraxic patients show a decreased regional blood flow measured by SPECT (Stamenova et al. 2015) or hypometabolism measured by PET (Frasson et al. 1998; Peigneux et al. 2001) in fronto-parietal areas that could only partly be explained by structural abnormalities. An EEG study (Wheaton et al. 2008) found a lack of beta-band coherence between left parietal and left premotor areas but instead an increased coherence in right fronto-parietal regions in 5 apraxic patients with left hemispheric damage due to corticobasal degeneration or stroke during pantomime of tool use. Although limited by the high variability in patients regarding lesion type and location as well as the small sample size, these results suggest a disturbance of the left-hemispheric fronto-parietal praxis network in apraxic patients.

Functional imaging studies in PD have so far focused on basal motor abilities revealing aberrant activations in the motor network (Catalan et al. 1999; Sabatini et al. 2000; Haslinger et al. 2001; Buhmann et al. 2003; Wu and Hallett 2005; Ukmar et al. 2006; Yu et al. 2007; Foki et al. 2015; see Tessitore et al. 2014 for review). PD patients showed increased activation relative to controls in the primary motor cortex, lateral premotor areas, and in the cerebellum in studies comparing simple finger or hand movements with rest conditions (Sabatini et al. 2000; Haslinger et al. 2001; Ukmar et al. 2006; Yu et al. 2007). Studies contrasting complex vs. simple motor tasks reported additional hyperactivation in parietal areas (Catalan et al. 1999; Wu and Hallett 2005). Hypoactivation during simple motor tasks was found in PD patients OFF in the SMA (Haslinger et al. 2001; Buhmann et al. 2003; Yu et al. 2007) and in the primary motor cortex in drug-naïve patients (Buhmann et al. 2003).

While these studies shed light on the pathophysiology of motor dysfunctions in PD, they cannot explain why a substantial part of patients with PD show additional praxis difficulties. By definition, apraxia cannot be attributed to basal motor or sensory dysfunctions. As outlined above praxis functions depend on the integrity of a widespread cortical network subserving several integrative and cognitive functions such as object recognition, sensorimotor and visuospatial integration, temporal and spatial organization of movements, and conceptual understanding of gestures and objects (Bieńkiewicz et al. 2014). Lesion studies indicate that impairment of each of this functions and corresponding brain regions can lead or contribute to apraxic symptoms. While apraxia resulting from stroke or tumor can be clearly attributed to a circumscribed lesion, defining the neuropathological basis for praxis deficits in PD is more challenging. Since PD is not only associated with basal ganglia dysfunction but also with a progressive cortical neurodegeneration, a number of candidate regions for producing apraxic symptoms have to be taken into account. Behavioral studies demonstrated that PD patients with apraxia primarily show impairments of the spatial organization of gestural movements that is proposed to depend on the integration of occipital, parietal, and frontal activation (Leiguarda et al. 1997; Binkofski and Buxbaum 2013). It’s likely that patients with clinically overt apraxia show impaired functional activation in these areas. Since apraxic symptoms in PD rather evolve than occur (compared to stroke) there is a chance for detecting functional aberrations of praxis subserving regions even before the symptoms become clinically apparent and obstructive for individual functioning and wellbeing. Vanbellingen et al. (2012) already demonstrated that the prevalence of apraxia increases with disease severity in patients with PD implying that patients in subclinical apraxia stages may already show functional aberrations. Therefore, we selected patients in early stages of PD without a diagnosis of apraxia according to cut-off values of accepted clinical scales and subjected them to a praxis sensitive fMRI task. The De Renzi Ideomotor apraxia test (De Renzi et al. 1980) is a widely accepted clinical apraxia score which defines existence of apraxia with score values below 62. Accordingly, values above 62 may well indicate some “subclinical” apraxic deficits and the scale may be used to correlate imaging findings with score values. We also investigated patients ON and OFF dopaminergic therapy to test if antiparkinson medication affects praxis abilities and related brain activation. Our primary hypothesis was to find activation differences in PD patients compared to healthy controls in praxis related brain areas. Further, we assumed that praxis related activations correlate with praxis test scores on a single-subject level.

## Methods

### Participants

Fourteen patients with PD, recruited at the Department of Neurology of the Medical University of Vienna, entered this study. One patient was not able to perform the experiment in the OFF state and was thus excluded from final analysis. For the resulting 13 patients (six female, mean 58.7 years), age at disease onset was 54.2 years on average and mean disease duration was 6.3 years (Table 1). To be included, patients had to be right-handed as assessed with the Edinburgh Handedness Inventory (Salmaso and Longoni 1985), not older than 85 years of age, and show mild parkinsonian symptoms (Hoehn and Yahr stage 1–2) and no apraxia as determined by standard clinical evaluations ON dopaminergic therapy (see 2.2.). None of the patients showed a coexistence of other neurological diseases, significant head tremor, disabling rest or action tremor according to the UPDRS III (action tremor > 2), history of psychosis, cognitive impairment according to the Mini-Mental State Examination (MMSE, Folstein et al. 1975) using a cut-off value of < 26 (Kukull et al. 1994; Pezzotti et al. 2008), abnormal sensory functions as determined by clinical examination, or abnormal pentagon drawing in the MMSE. 14 healthy control subjects (HC) matched for age and gender (five female, mean 57.4 years) were recruited from the general population. Control subjects had a normal neurological and psychiatric status without any history of CNS disease or a first grade relative with a primary movement disorder. The study was approved by the ethics committee of the Medical University of Vienna and all participants gave their written informed consents according to the Declaration of Helsinki.

**Table 1:** Subject characteristics and behavioral data

	Patients (*N* = 13)		HC (*N* = 14)
	ON	OFF	
Gender	6 female, 7 male		5 female, 9 male
Age	58.7 (13.0) years		57.4 (9.8) years
Age at disease onset (years)	54.2 (12.4)		
Disease duration (years)	6.3 (4.7)		
UPDRS III	22.5 (7.8) *	30.2 (12.2) *	
^a^Bradykinesia-Score	4.5 (1.3) *	5.8 (2.2) *	
No. Patients H&Y 2/2.5/3	8/5/0	5/7/1	
De Renzi Demonstration-of-Use score	20.0 (0.0)	20.0 (0.0)	20.0 (0.0)
De Renzi Ideomotor apraxia score (Cut-Off Value for Apraxia = 62)	70.9 (1.3)	70.2 (1.6)**	71.6 (0.6)**
Pantomime Rating	2.6 (0.4)	2.5 (0.5) **	2.7 (0.3) **
Finger taps per 20 s	21.1 (2.5)	22.1 (3.8)	20.5 (2.5)

Data as mean (standard deviation)

*UPDRS* Unified Parkinson’s Disease Rating Scale, *H&Y* Hoehn and Yahr stage

^a^Individual cumulative bradykinesia indices of the right hand: sum of scores for items 23–25 of motor UPDRS

*Significant difference between patients ON and OFF (*P* < .05)

**Significant difference comparing healthy controls (*HC*) and patients (*P* < .05)

### Clinical evaluations

All participants performed the De Renzi Ideomotor apraxia test (De Renzi et al. 1980) and the De Renzi Demonstration-of-Use test (De Renzi and Lucchelli 1988) before the fMRI experiments. In addition, patients underwent a neurological examination including Hoehn and Yahr scale, UPDRS III (including action tremor item 21 and bradykinesia index), and MMSE before each measurement (ON and OFF).

### Experimental design

**Fig. 1:**
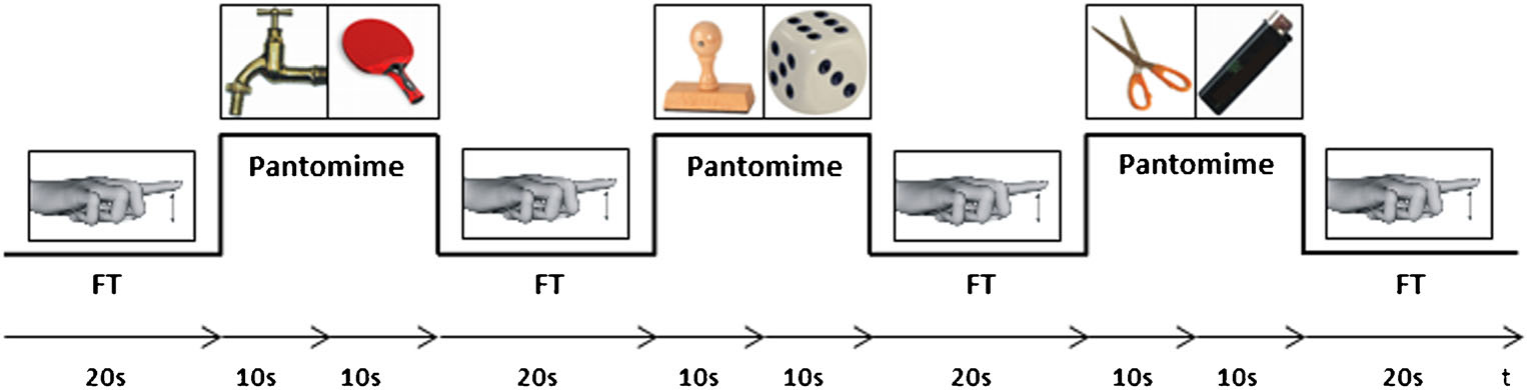
Time course of a single fMRI run with 4 reference (finger tapping, FT) and 3 pantomime periods (each lasting 20 s). During one pantomime period two pictures of objects were consecutively presented for 10 s each, while the image indicating finger tapping was visible for 20 s

During fMRI participants performed a pantomime task in which they imitated the use of visually presented objects with their right hand. This task has previously been shown to be highly sensitive for praxis functions (Heilman and Rothi 1993; Goldenberg and Spatt 2009). In accordance with previous studies (Choi et al. 2001; Ohgami et al. 2004; Foki et al. 2015) effects resulting from basic motor skills (e.g. bradykinesia) were minimized by applying standardized 1 Hz right index finger tapping (FT) as reference task. The slow paced finger tapping could be reliably performed by mildly to moderately affected patients with PD (ON and OFF). The block-designed fMRI experiment consisted of 10 runs with four reference (FT) and three pantomime periods (each lasting 20 s). During one pantomime period two pictures of objects (e.g. a tap followed by a table tennis racket) were presented consecutively (10 s each), while the image indicating FT was visible throughout the whole FT period (Fig. 1). Within every run, six different pictures of objects were shown. Overall, 30 objects were used twice within the 10 runs, but never consecutively. Participants were not able to see their actual pantomime gestures or finger tapping. Intrascan pantomime performance was semiquantitatively rated by the experimenter according to a rating suggested by Leiguarda et al. (1997) for transitive movements: three points were given for a gesture appropriate for the actual object, two points for a gesture that resembled the correct one but included temporal or spatial errors, one point for a movement with a weak resemblance to the appropriate gesture. No points were noted when the gesture was too wrong or too incomplete to be recognizable. The number of finger taps per reference block was recorded as well, with feedback given right after each run in case of inappropriate FT frequency. Each patient was measured twice, once with individually optimized dopaminergic medication (ON) and once without dopaminergic medication (OFF). Patients in the OFF state were measured at least 12 h after their last dopaminergic medication, or in case of extended-release preparations after at least 48 h. Scans ON and OFF were conducted in a randomized order (seven patients performed ON first, six OFF first) within 14 days.

### Data acquisition

Images were acquired with a 3 Tesla TIM-TRIO system (Siemens Healthcare, Erlangen, Germany) using a 32 channel Siemens head coil. 34 slices were aligned with the AC-PC plane, covering the whole brain (slice-thickness 3 mm, matrix 128*128, FOV 230*230 mm, voxel size 1.8*1.8*3.0 mm). To minimize head motion artefacts, individually constructed plaster cast helmets were used (Edward et al. 2000). A 2D single shot EPI-sequence was used with TE 35 ms, TR 2500 ms, parallel imaging with GRAPPA (IPAD = 2). Each of the 10 fMRI runs consisted of 56 volumes and lasted 140 s (preceded by 10 s of dummy scans).

### MR data processing

Image preprocessing and statistical analysis at individual and group level were performed using SPM8 (Wellcome Department of Imaging Neuroscience, London, UK; http://www.fil.ion.ucl.ac.uk/spm). To reduce residual small-scale motion, all runs were realigned to the first scan using default settings (except for factor “Quality” set to 1). Realigned data were then normalized to MNI space and smoothed with a 8 × 8 × 8 mm full width at half maximum (FWHM) Gaussian kernel. First level statistical analysis was performed with a fixed effects analysis (SPM default settings). Blood-oxygen-level dependent (BOLD) responses were modeled by a fixed response boxcar function convolved with the canonical hemodynamic response. As four patients were not able to accomplish all 10 runs, group analysis was performed with individual SPM t-maps weighted according to run number. Group activations were thresholded at a familywise error rate (FWE) adjusted *P* < 0.05 with an extend cluster size (k) of 50 voxels. Contrasts between patients ON or OFF and HC were calculated using unpaired t-tests, the comparison between ON and OFF state was done by a paired *t*-test. Group comparisons were thresholded at *P* < 0.001 uncorrected with a 50-voxel extent threshold. The probabilistic Harvard-Oxford cortical and subcortical Structural atlases (http://www.cma.mgh.harvard.edu/) were used to assign functional activation to anatomical structures.

### Region of interest analysis

Regions of the praxis network that showed functional aberrations in PD patients compared to HC on group level were subjected to a region of interest (ROI) analysis. As probabilistic atlases of macroscopic anatomy are more favourable for ROI analysis than deterministic atlases such as the AAL atlas or the Talairach atlas (Poldrack 2007), ROIs were generated by using the probabilistic Harvard-Oxford Cortical Structural Atlas implemented in FSL (FMRIB Software Library v5.0; http://www.fmrib.ox.ac.uk/fsl/) and were transferred to the individual SPM t-maps of each participant to determine the mean T-value at an uncorrected threshold of *P* < 0.001. The mean T-values within the ROIs were compared between groups by using appropriate t-tests (unpaired t-tests for ON vs. HC and OFF vs. HC, paired t-tests for the comparison between ON and OFF; two-tailed). To test the relation between activation within the ROIs and behavioral data, mean T-value of each ROI and clinical scores (Hoehn and Yahr scale, UPDRS-score, action tremor score, bradykinesia index, MMSE score, De Renzi Ideomotor apraxia score) entered Spearman’s rank correlation analysis (SPSS version 20). Spearman’s rank correlation was favored over Pearson’s correlation since it is more reliable in small data sets and can be applied to non-normal data (Elliott and Woodward 2007).

### Statistical analysis of behavioral data

For every measurement, pantomime performance ratings and finger tapping per period were averaged. Intrascan behavioral performance and clinical measures (Hoehn and Yahr scale, UPDRS-score, action tremor score, bradykinesia index, MMSE score, De Renzi Ideomotor apraxia score) were tested for normality with a K-S-test (SPSS version 20). For the comparison between patients ON and OFF paired t-tests (two-tailed, alpha level set at *P* < 0.05) were applied for all behavioral and clinical scores. Differences between patients ON vs. HC and patients OFF vs. HC in these scores were tested by using unpaired t-tests (two-tailed, alpha level set at *P* < 0.05). The De Renzi Ideomotor apraxia scores entered Mann–Whitney-U-tests for the comparison between HC and patients as these data were not normally distributed.

## Results

### Clinical evaluation and behavioral performance

Subject characteristics and data of the clinical assessments and the behavioral performance are summarized in Table 1. PD patients and HC did not significantly differ according to their age (*P* = 0.765). Patients OFF exhibited significantly worse motor signs (UPDRS III-score: *P* = .022) including bradykinesia (sum of scores for items 23–25 of motor UPDRS, *P* = 0.016) than patients ON. Hoehn and Yahr stage increased 0.5 points in four patients in the OFF state, while for the other nine patients Hoehn and Yahr stages remained stable across ON and OFF states. All participants achieved the maximum point score (20) in the De Renzi Demonstration-of-Use Test. In the De Renzi Ideomotor apraxia test 7 of the HC (median = 72), 5 of the patients ON (median = 71.5), but only 1 of the patients OFF (median = 70.5) reached the maximal score of 72. All controls scored ≥ 70 (range 70–72) while 2 of the patients ON (range 68–72) and 4 of the patients OFF (range 67–72) scored below 70. None of the patients showed apraxic symptoms according to the De Renzi Ideomotor apraxia score (cutoff value: 62) and there was no significant difference between ON and OFF state in this score (*P* = 0.162). However, patients OFF (*P* = 0.008) but not patients ON (*P* = 0.205) had significantly lower De Renzi Ideomotor apraxia scores than controls. In addition, patients OFF exhibited a decreased intrascan pantomime performance compared to HC (patients ON vs. HC: *P* = 0.132; patients OFF vs. HC: *P* = 0.043; patients ON vs. OFF: *P* = 0.254). Mean FT frequency was similar across all groups (patients ON vs. HC: *P* = 0.565; patients OFF vs. HC: *P* = 0.219; patients ON vs. OFF: *P* = 0.256).

### Functional imaging

#### Group activation for HC, patients ON and OFF

**Fig. 2:**
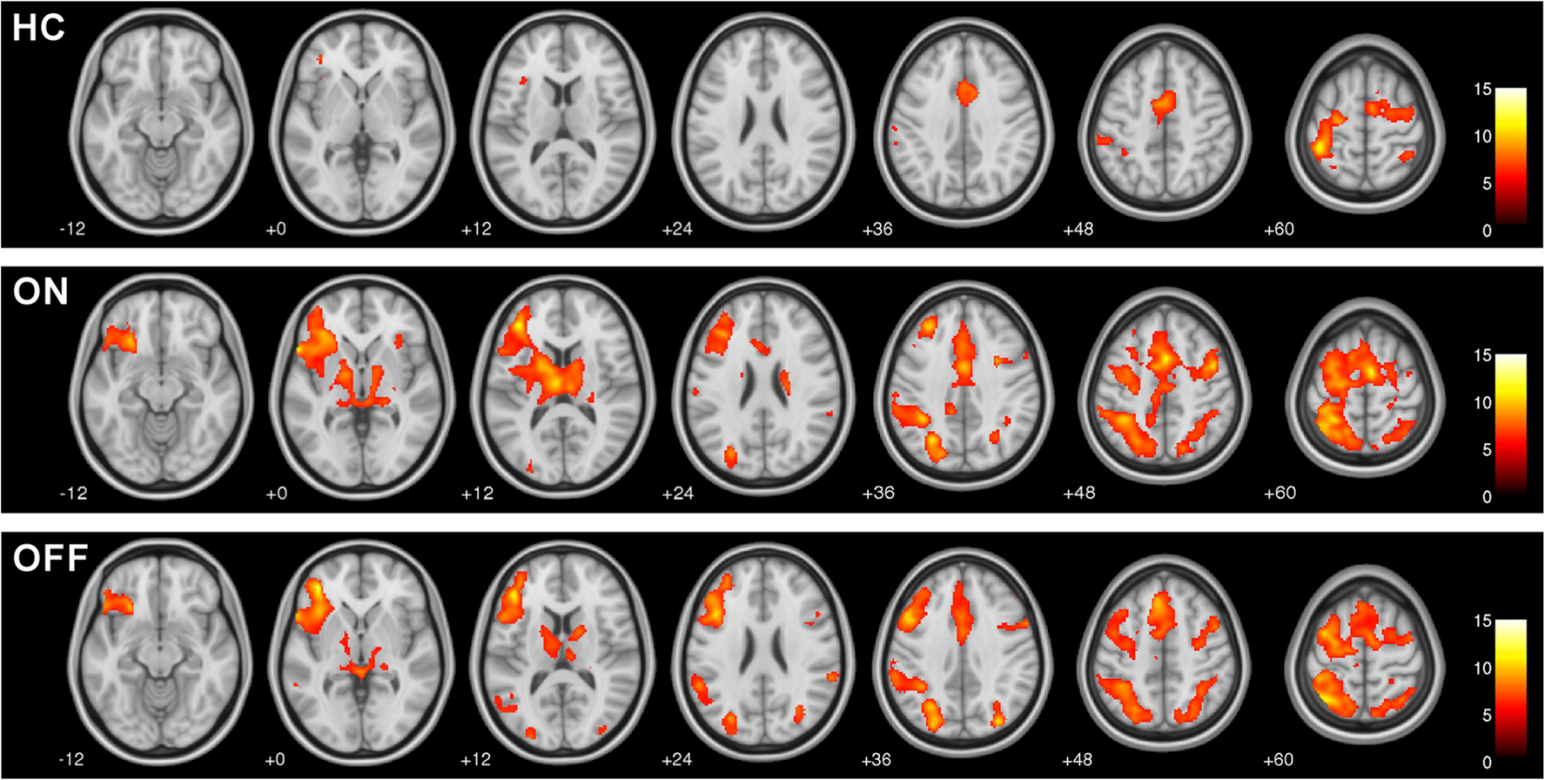
Group activation for praxis related fMRI signals for healthy controls (*HC*), patients ON and OFF dopaminergic medication (Pantomime vs. finger tapping; FWE corrected, *P* < 0.05, k = 50). All experimental groups showed bilateral, but predominantly left-hemispheric praxis related activation in fronto-parietal areas. Patients in both states exhibited stronger and more widespread activation, in particular in bilateral parietal regions and left frontal areas

In HC fMRI revealed significant pantomime related signal modulations in the bilateral superior parietal lobe, left supramarginal gyrus, left postcentral gyrus and bilateral motor cortex encompassing the supplementary motor area, cingulate gyrus, and superior frontal areas, as well as smaller clusters in left prefrontal regions (*P* < 0.05, FWE corrected, k = 50; Fig. 2, upper row). Patients in the ON and OFF state exhibited clearly stronger and more widespread pantomime related functional activation in regions also observed in HC, in particular in bilateral parietal regions and left frontal areas (Fig. 2, middle and lower row). Parietal activation in patients included bilateral activation of the supramarginal gyrus and superior parietal lobe as well as left lateralized activation in the angular gyrus and in the precuneus. As HC, patients displayed activation in left prefrontal areas (frontal pole, frontal orbital cortex, frontal operculum cortex), in bilateral superior frontal and cingulate gyri as well as in bilateral motor regions (precentral gyrus, supplementary motor area). However, activation in inferior and middle frontal gyri was found in patients only. Additionally, patients showed significant activation in the insular cortex, in the thalamus and in bilateral occipital areas that was absent in HC. Activation in the basal ganglia (bilateral putamen and pallidum, right caudate) was exclusively observed in patients ON. Peak activation with highest T-values, cluster size, and MNI coordinates are listed in Table 2.

**Table 2:** Praxis related peak activation in HC, Patients ON and Patients OFF

Location	HC					Patients ON					Patients OFF				
	T	N	MNI Coordinates			T	N	MNI Coordinates			T	N	MNI Coordinates		
			X	Y	Z			X	Y	Z			X	Y	Z
Frontal Pole L	7.97	67	−36	40	−4	11.67	27,209	−38	38	14	12.74	14,669	−38	42	0
Frontal Orbital Cortex L	5.79	93	−36	24	−2	9.91	27,209	−30	28	−4	9.78	14,669	−26	24	−12
Frontal Orbital Cortex R						9.54	94	50	22	−8					
Frontal Operculum Cortex L	7.1	93	−34	20	6	8.85	27,209	−42	16	2					
IFG L						11.06	27,209	−58	18	0	10.5	14,669	−42	28	18
IFG R											6.20	14,669	50	18	22
MFG L						10.31	27,209	−36	36	32	14.32	14,669	−46	14	32
MFG R						10.04	27,209	30	6	38	8.63	105	48	34	30
SFG L	7.48	1933	−20	−4	72	10.76	27,209	−14	−12	70	14.76	14,669	−14	−12	66
SFG R	8.66	2503	22	−8	64	8.32	27,209	12	−2	70	8.11	14,669	20	0	54
Precentral Gyrus L	9.86	1933	−16	−14	66	9.52	27,209	−22	−16	62	9.35	14,669	−26	−8	60
Precentral Gyrus R	8.44	2503	16	−12	64	10.73	27,209	44	2	48	11.04	14,669	14	−14	64
SMA L						10.65	27,209	−8	0	54	8.24	14,669	−4	−8	70
SMA R	9.97	2503	4	0	54	11.27	27,209	6	−4	60	9.09	14,669	6	4	68
Cingulate Gyrus L	9.37	2503	0	2	44	9.96	27,209	0	2	38	8.36	14,669	−2	16	40
Cingulate Gyrus R	8.63	2503	10	10	38	11.20	27,209	2	8	46					
Insular Cortex L						6.98	27,209	−36	0	12	9.81	14,669	−36	20	2
Insular Cortex R						7.35	121	34	20	4					
Angular Gyrus L						6.62	27,209	−50	−52	40	7.35	6171	−54	−60	20
Supramarginal Gyrus L	7.46	1933	−54	−36	44	10.47	27,209	−36	−46	38	9.4	6171	−38	−46	38
Supramarginal Gyrus R						6.99	64	66	−34	28	8.43	106	62	−38	26
SPL L	6.79	1933	−28	−58	64	11.69	27,209	−38	−50	54	10.73	6171	−38	−56	58
SPL R	7.4	200	38	−46	58	8.28	1697	34	−52	54	8.05	2258	34	−52	50
Precuneus L						7.39	27,209	−8	−48	52	8.75	6171	−4	−58	72
Postcentral Gyrus L	12.09	1933	−42	−40	62	9.61	27,209	−38	−38	60					
Postcentral Gyrus R						7.5	82	26	−34	56	6.1	74	30	−38	56
Occipital Pole L						6.72	27,209	−28	−94	12	7.57	6171	−28	−94	12
LOC L						11.28	27,209	−28	−64	38	11.61	6171	−26	−76	30
LOC R						8.33	1697	16	−74	50	10.49	2258	32	−78	34
Putamen L						8.70	27,209	−26	18	2					
Putamen R						6.66	27,209	28	−16	4					
Pallidum L						9.41	27,209	−14	−6	2					
Pallidum R						7.90	27,209	14	−6	−2					
Caudate R						8.16	27,209	12	2	12					
Thalamus L						10.65	27,209	−6	−14	12	7.82	1637	−20	−30	−4
Thalamus R						8.81	27,209	8	−16	12	7.32	1637	8	−20	12

Local maxima of task specific activations (pantomime vs. finger tapping) for all groups with T-values, cluster size, and MNI coordinates (*P* < 0.05, FWE corrected, T > 5.73, k = 50). For location assignment the Harvard-Oxford cortical and subcortical structural atlases were used

*IFG* inferior frontal gyrus, *MFG* middle frontal gyrus, *SFG* superior frontal gyrus, *SMA* Supplementary motor area, *SPL* superior parietal lobe, *LOC* lateral occipital cortex, *L* left, *R* right

#### Whole brain group comparisons

**Fig. 3:**
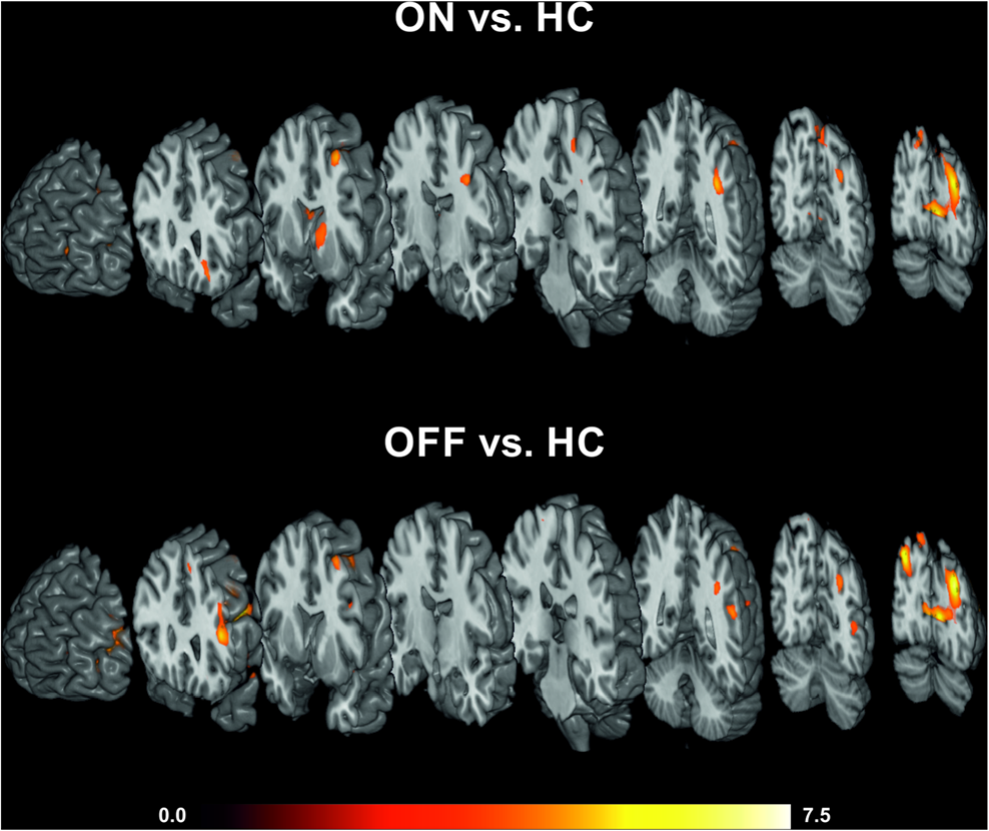
Significantly increased praxis related activation in patients ON and OFF compared to healthy controls (*HC*; *P* < 0.001, uncorrected, k = 50). Patients ON and OFF showed hyperactivation in left fronto-parietal and bilateral occipital areas. Differential hyperactivation between the ON and OFF state were found in the frontal orbital cortex, the inferior frontal gyrus, basal ganglia, precentral gyrus, angular gyrus, and superior parietal lobe (compare contrasts ON vs. HC and OFF vs. HC)

The results of the statistical contrasts of task-related activation for patients ON vs. HC and patients OFF vs. HC are depicted in Fig. 3 and Table 3 (*P* < 0.001, uncorrected, k = 50). Generally, a hyperactivation of the praxis network was found in patients. Compared to HC, patients in both states showed significantly higher activation in left frontal areas (middle and superior frontal gyri, frontal pole). Further, patients ON displayed hyperactivation in left frontal orbital cortex and left precentral gyrus, whereas patients OFF exhibited hyperactivation in the left inferior frontal gyrus. In patients ON left parietal hyperactivation encompassed the supramarginal gyrus, the superior parietal lobe, and the precuneus while in patients OFF the parietal hyperactivation was limited to the left inferior parietal lobe (including angular gyrus). Both patients ON and OFF displayed a bilateral hyperactivation in occipital regions reaching from primary visual areas (intracalcarine cortex, occipital pole) to superior parts of the lateral occipital cortex. Additional local maxima were also found in the left striatum (caudate nucleus and putamen) and in the left thalamus in patients ON. At the given threshold (*P* < 0.001, uncorrected, k = 50) no region was found with significantly lower praxis related activation in patients (both OFF and ON) compared to HC. A direct contrast between patients ON and OFF did not reveal any significant activation differences at this threshold too.

**Table 3:** Location of local maxima for the contrasts between patients and HC

Location	ON vs. HC					OFF vs. HC				
	T-value	Cluster size	MNI-Coordinates			T-value	Cluster size	MNI-Coordinates		
			X	Y	Z			X	Y	Z
Frontal Pole L	4.99	278	−40	38	14	4.54	1412	−40	42	2
	4.76	50	−28	56	8					
Frontal Orbital Cortex L	4.67	818	−26	24	−10					
IFG L						5.47	1412	−44	34	12
MFG L	5.00	710	−32	10	58	7.22	1412	−48	16	32
	4.13	278	−30	34	40	4.71	254	−36	2	58
SFG L	3.72	278	−26	32	52	4.13	51	−6	26	52
Precentral Gyrus L	4.86	710	−36	−8	42					
Angular Gyrus L						3.86	195	−58	−54	30
Supramarginal Gyrus L	5.14	2723	−36	−46	36	4.60	195	−54	−48	26
						4.27	65	−36	−48	36
SPL L	3.92	2723	−16	−58	56					
Precuneus L	4.35	2723	−2	−72	54					
Intracalcerine Cortex L	5.79	2723	−8	−82	6	5.84	2323	−10	−80	6
Intracalcerine Cortex R	4.84	2723	6	−76	4	5.00	2323	4	−78	6
Occipital Pole L	6.82	2723	−22	−96	18	6.47	2323	−26	−94	16
LOC superior division L	6.38	2723	−28	−76	26	7.50	2323	−26	−76	30
LOC inferior division L						4.01	81	−44	−70	12
LOC superior division R	4.45	104	12	−82	48	6.14	650	32	−78	36
	3.91	77	24	−62	50	4.36	152	14	−80	50
						4.16	52	34	−66	58
Caudate Nucleus L	5.15	818	−16	22	−6					
Pallidum L	3.61	818	−14	−6	2					
Thalamus L	3.69	818	−4	−14	12					

Local peak activation with T-value, cluster size, and MNI coordinates for the contrast patients ON vs. HC and patients OFF vs. HC (*P* < 0.001, uncorrected, T > 3.45). For location assignment the Harvard-Oxford Structural Atlas was used

*IFG* inferior frontal gyrus, *MFG* middle frontal gyrus, *SFG* superior frontal gyrus, *SPL* superior parietal lobe, *LOC* lateral occipital cortex, *L* left, *R* right

#### Region of interest analysis

The functional group analyses demonstrated hyperactivation in core areas of the described praxis network in PD patients. Patients displayed hyperactivation of left parietal areas (more pronounced in the ON state) and in left inferior and middle frontal regions. Therefore, we generated a left parietal ROI including inferior and superior parietal areas and left frontal ROI encompassing inferior and middle frontal gyri and analyzed individual pantomime related activation within these two ROIs. Mean T-values within the parietal and frontal ROI were higher in patients, however, a significant difference was found in the frontal ROI only (Table 4) indicating that frontal hyperactivation was disproportionately high compared to parietal areas. When correlating brain activation with praxis test scores, the mean T-values of the frontal ROI showed a trend towards a negative correlation with the De Renzi Ideomotor apraxia scores (R = −.297, *P* = .074). In contrast, there was a slight non-significant positive relation between the mean T-values of the parietal ROI and this score (R = .111, *P* = .514). To further test this apparent inverse relationship between frontal and parietal activation regarding the correlation with the De Renzi Ideomotor apraxia score we generated a left fronto-parietal index (FPI) for every participant by using this formula: (mean T-value of the left frontal ROI - mean T-value of the left parietal ROI) divided by (mean T-value of the left frontal ROI + mean T-value of the left parietal ROI). Hence, a positive FPI value indicates frontal activation dominance while a negative value points to parietal dominance. Statistical comparisons between groups revealed significantly higher FPI values for patients ON and OFF compared to HC (Table 4). There were no significant differences between patients ON and OFF regarding mean T-Value within the frontal (*P* = .979) or parietal ROI (*P* = .265) nor regarding the FPI (*P* = .390). A Spearman’s rank correlation analysis between FPI values and De Renzi Ideomotor apraxia scores of all measurements (patients ON and OFF, HC) revealed a negative correlation (R = −.399, *P* = .014) meaning that a frontal activation dominance was related to lower praxis scores. No other clinical score, behavioral or demographic measure was significantly correlated with the FPI. To adjust for possible confounding effects of parkinsonian symptoms, an additional Spearman’s rank partial correlation analysis between FPI values and De Renzi Ideomotor apraxia scores with the UPDRS-score as control variables was performed. As HCs were not subjected to the UPDRS, this analysis was restricted to patient data (ON and OFF). Again, a negative correlation between the FPI and the De Renzi score was found (R = −.342, *P* = .102), that - probably due to the smaller sample size - did not reach statistical significance.

**Table 4:** Mean T-Values within ROIs and fronto-parietal index for all groups

	HC (*N* = 14)	ON (*N* = 13)		OFF (*N* = 13)	
	Mean (SD)	Mean (SD)	*P* value	Mean (SD)	*P* value
Frontal ROI	5.28(1.51)	8.14(2.14)	.000	8.11(3.06)	.008
Parietal ROI	7.09(2.06)	8.17(1.45)	.128	7.45(2.18)	.661
Fronto-parietal Index	−0.14(0.15)	−0.01(0.15)	.027	0.03(0.09)	.001

Mean and standard deviation (*SD*) of mean T-Values within the left-hemispheric regions of interest (*ROI*) and corresponding fronto-parietal Index for all groups with *P* -values derived from unpaired t-tests between patients and HC

**Fig. 4:**
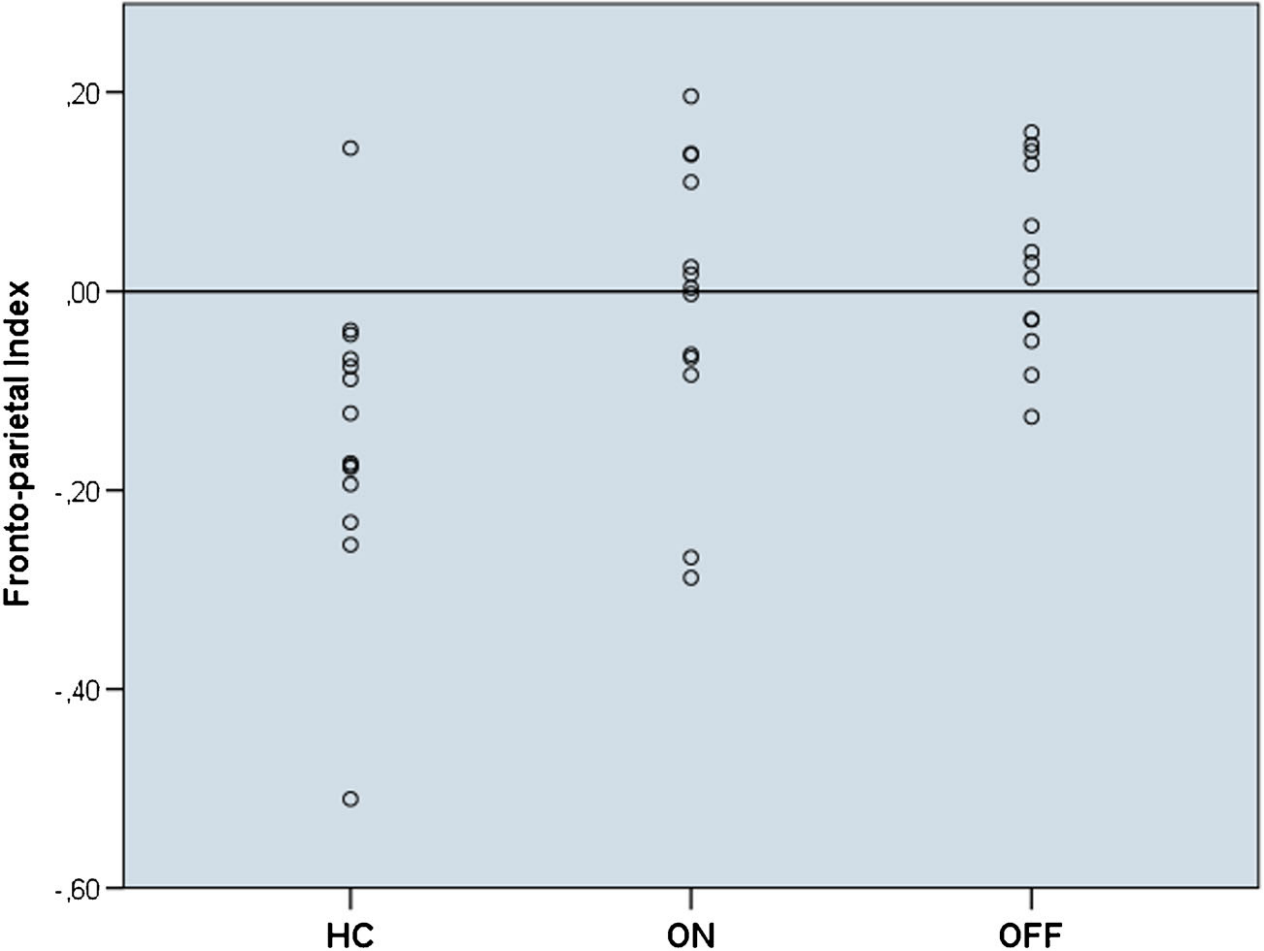
Fronto-parietal index values for all participants generated from the individual mean T-values within frontal and parietal ROIs. A positive fronto-parietal index value indicates frontal activation dominance as displayed in 62 % of the patients (ON and OFF) while a negative value points to parietal dominance as shown by 94 % of the healthy controls (*HC*)

To test whether the FPI can be used for distinguishing PD patients as a risk group for apraxia and HC the sensitivity and specificity for this index was calculated. The sensitivity was defined as the probability of a positive test result (FPI ≥ 0) given the disease is present (patients with FPI ≥ 0 / all patients) and was 62 % (16 out of 26 patient measurements displayed an index value ≥ 0). The specificity was defined as the probability of a true negative result (HC with FPI < 0 / all HC) and was 93 % as 13 of 14 controls showed a negative index value. Parietal dominance (FPI < 0) was present in 5 patients in both states, but 16 of 17 cases with FPI ≥ 0 indicating frontal activation dominance were patients (8 patients ON and 8 patients OFF, Fig. 4).

## Discussion

### Major results and relation to previous literature

Patients with PD have an increased risk for developing apraxic symptoms in the course of the disease (Vanbellingen et al. 2012). As in other movement disorders, praxis deficits may be superimposed on elementary motor impairments contributing to defective manual skill and hamper activities of daily living (Zadikoff and Lang 2005). However, up to now functional imaging studies in PD have only addressed basal motor abilities (Catalan et al. 1999; Sabatini et al. 2000; Haslinger et al. 2001; Buhmann et al. 2003; Wu and Hallett 2005; Ukmar et al. 2006; Yu et al. 2007; Schwingenschuh et al. 2013; Martinu et al. 2014; Foki et al. 2015; Wu et al. 2015; see Tessitore et al. 2014 for review). This exploratory fMRI study of praxis functions in a clinical population provides first data about an early compensatory behavior of the praxis network and a possible individual imaging marker for incipient apraxia.

#### Hyperactivation in the praxis network in PD patients

According to previous lesion studies (Buxbaum et al. 2003, 2007, 2014; Goldenberg et al. 2007; Goldenberg and Spatt 2009; Randerath et al. 2010; Hermsdörfer et al. 2013; Manuel et al. 2013) and functional imaging literature (Choi et al. 2001; Ohgami et al. 2004; Fridman et al. 2006; Imazu et al. 2007; Vingerhoets et al. 2011; Brandi et al. 2014; Mäki-Marttunen et al. 2014; Niessen et al. 2014; Vry et al. 2015) praxis functions are organized in a widespread left-hemispheric network with parietal and frontal core areas. Corresponding to the majority of the functional imaging studies in healthy subjects we found bilateral, but predominantly left-hemispheric pantomime related activation in fronto-parietal areas in all experimental groups (HC, patients ON and OFF dopaminergic medication). Compared to HC, patients in both states exhibited hyperactivation in key areas of the praxis network, namely the left inferior parietal and left middle frontal areas. The inferior parietal lobe is most consistently found to be associated with praxis (dys)functions and is thought to contain long-term representations of skilled object-related actions (Heilman et al. 1982; Binkofski and Buxbaum 2013). The middle frontal gyrus is commonly reported to be co-activated with parietal areas in functional imaging studies in normal subjects and lesions in this area or the underlying white matter were found to produce apraxia (Manuel et al. 2013; Buxbaum et al. 2014). The middle frontal gyrus - in particular its dorsolateral prefrontal part - is related to executive functions (Goldenberg and Spatt 2009) like selecting among competing responses (Ridderinkhof et al. 2004). Hyperactivation in fronto-parietal areas were clearly lateralized to the left hemisphere in group contrast (ON vs. HC and OFF vs. HC) supporting the relevance of the left hemisphere for praxis functions.

Hyperactivation in patients was also found in bilateral occipital areas involving primary visual cortex and superior parts of the lateral occipital cortex. The lateral occipital cortex plays an important role in object recognition (Grill-Spector et al. 2001) and its superior parts form the starting point of the dorso-dorsal pathway that is relevant for monitoring the online control of objects (Binkofski and Buxbaum 2013; Brandi et al. 2014). Thus, hyperactivation of visual areas might be a hint for a stronger dependence on the visually presented cue to perform the pantomime task in PD patients.

The capability of parkinsonian brains to counteract behavioral motor deficits via local neuronal hyperactivation has already been demonstrated in fMRI studies addressing basal motor abilities (Sabatini et al. 2000; Haslinger et al. 2001; Wu and Hallett 2005; Ukmar et al. 2006; Yu et al. 2007; Foki et al. 2015). Functional hyperactivation can be observed in various neurological diseases including stroke, epilepsy, tumor, and multiple sclerosis (Beisteiner and Matt 2015) as well as in prodromal phases of Alzheimer’s disease (Dickerson and Sperling 2008; Sugarman et al. 2012). Such hyperactivation is commonly interpreted as a compensatory mechanism that allows a relatively normal behavioral performance (Dickerson and Sperling 2008; Sugarman et al. 2012). As none of the patients was classified as apraxic according to the De Renzi Ideomotor apraxia score, our findings most likely represent successful compensatory hyperactivation in regions typically related to praxis functions.

#### Frontal compensation of parietal dysfunctions

As evident from prevalence figures of apraxia in PD (40 % in Hoehn and Yahr stage 4, Vanbellingen et al. 2012) probably not all of the patients investigated will develop apraxia. This might be due to the fact that a part of the patients are not prone to apraxia at all but there also might be compensatory mechanisms like hyperactivation that can be sustained throughout the course of the disease in some patients. The current finding of hyperactivations in praxis networks demonstrates that brain activation in this sample deviates from normal functioning and indicates that patients need more effort and/or more resources to accomplish a praxis task.

The FPI indicating the individual relation between frontal and parietal activation was significantly higher in patients in both states compared to HC and confirmed frontal activation dominance in 62 % of the patients and parietal dominance in 93 % of the controls. Frontal activation dominance was only found in one control subject resulting in the high specificity (93 %) of the FPI. However, the rather low sensitivity (62 %) of the FPI to distinguish between PD patients as a risk group and HC mirrors the fact that probably only a part of the patients investigated will develop apraxia. Future studies that test the sensitivity and specificity of the FPI for clinically apparent apraxia will possibly result in higher values for sensitivity. Although we only included patients scoring above a cut-off value for clinical apraxia (thus limiting the variability in this measure) a significantly negative correlation between the FPI and De Renzi Ideomotor apraxia score was found. Importantly, no other clinical measure showed a significant correlation with the FPI meaning a frontal dominance was associated with lower praxis abilities but not with parkinsonian symptoms, behavioral or demographic measures. This result points to an increased frontal activation that might compensate for already disturbed parietal functions (Goldenberg and Spatt 2009).

However, the observed hyperactivation in large parts of the left fronto-parietal praxis network as a putative compensatory mechanism in PD patients might not be maintained over a longer period of time. In other neurodegenerative diseases like Alzheimer’s disease the phase of compensatory hyperactivation is suggested to be followed by breakdown of these networks behaviorally apparent as a loss of function and neurophysiologically characterized by a recruitment of additional brain regions and/or underactivation in respective networks (Dickerson and Sperling 2008; O’Brien et al. 2010; Sugarman et al. 2012). The lack of hyperactivation in the superior parietal lobe in patients OFF might point to this development since they had significantly lower clinical apraxia and intrascan pantomime performance scores than HC. Thus, the altered parietal activation in clinically non-apraxic patients may be followed by parietal hypoactivation in patients that actually develop apraxic symptoms. Therefore, it’s likely that the fronto-parietal imbalance aggravates as apraxia becomes clinically overt making the FPI even more sensitive. As it is difficult to isolate the apraxia component from other compromised motor components in pure clinical neurological examinations (Vanbellingen et al. 2012), such an objective apraxia marker would be helpful in PD and other movement disorders. Indeed, the FPI is based on individual brain activation data and its generation is thus independent from performance evaluations. Thus, we suggest the FPI as a candidate indicator for an ongoing process of fronto-parietal dysfunction that – when compensatory mechanisms fail – leads to apraxia.

#### Dopaminergic modulation via striato-cortical connections

In line with previous findings (Leiguarda et al. 1997) we found no significant difference in praxis performance scores between patients ON and OFF medication. A direct contrast between the ON and OFF state regarding brain activation did not reach significance at the chosen threshold too. However, there are some distinct and noticeable differences between patients ON and OFF relative to HC. The striatum was found to be overactivated in patients ON only, which is likely to be induced by dopaminergic medication (Schwingenschuh et al. 2013; Martinu et al. 2014). In addition, we found hyperactivation in the left superior parietal lobe and the left frontal orbital cortex in patients ON but not in patients OFF. Besides the hyperactivation in the primary motor cortex as also reported in previous studies (Buhmann et al. 2003; Foki et al. 2015) dopaminergic therapy seems to elevate activation in areas involved in executive control (frontal orbital cortex; Bryden and Roesch 2015) and in online monitoring of movements (superior parietal lobe; Binkofski and Buxbaum 2013) possibly via connections to the overactivated striatum (Jarbo and Verstynen 2015). As - opposed to patients OFF - patients ON did not differ in the behavioral and clinical apraxia scores from HC, dopaminergic therapy might not only alleviate typical parkinsonian motor signs including bradykinesia but might also facilitate the performance of complex motor task such as pantomime. The parietal hyperactivation in patients OFF was restricted to inferior parietal areas, but here included not only the supramarginal gyrus (that was also overactivated in the ON state) but the angular gyrus as well. This extended inferior parietal hyperactivation might be associated with the hyperactivation found in the inferior frontal gyrus via the arcuate fascicle (Vry et al. 2015) subserving praxis functions related to the ventro-dorsal stream (Binkofski and Buxbaum 2013; Brandi et al. 2014). As patients OFF displayed significantly lower apraxia and pantomime performance scores than HC, this might indicate that hyperactivation of the ventro-dorsal stream found in patients OFF is not as successful as striato-fronto-parietal hyperactivation possibly induced by dopaminergic therapy. However, these hints for a dopaminergic effect on praxis functions require further investigation with a larger sample of patients with and without medication and with a broader spectrum of praxis abilities.

### Limitations and outlook

With pantomime of object use a paradigm was applied that has been shown to be sensitive for praxis functions (Heilman and Rothi 1993; Goldenberg and Spatt 2009). However, other tasks such as the actual use of objects or imitation of gestures might elicit other activation patterns and functional aberrations in PD patients than found in this study. Although we only included patients without cognitive impairment as measured by the MMSE with a quite stringent cut-off value we cannot rule out that lower praxis scores in patients may be caused by specific cognitive deficits not addressed by the MMSE. A more detailed neuropsychological testing of the patients would be required to test a possible relationship of praxis deficits with specific cognitive abilities (e.g. executive functions).

Certainly, the current findings are restricted to the patient population investigated in this study – patients in early stages of PD without clinically evident apraxia – and the validation of the functional imaging characteristics in particular the FPI as a potential marker for apraxia has yet to be done. Besides of the comparison of patients with and without clinically apparent apraxia, future studies should monitor patients longitudinally to identify which activation patterns are characteristic for patients that actually develop apraxia and whether the FPI can really detect them. Methods that probe functional (functional connectivity, dynamic causal modelling) and structural (diffusion tensor imaging) networks could thereby complement task-based brain activation data. A reliable identification of subjects at risk for apraxia – potentially by using the FPI as an early and objective marker – would allow early therapeutic intervention to delay negative effects of apraxia on activities of daily life.

In conclusion, our findings show that early PD patients already show characteristic signs of praxis network dysfunctions and rely on specific hyperactivation to avoid clinically evident apraxic symptoms. However, subclinical deficits correlated with an activation shift from left parietal to left frontal areas implying a potential use of the FPI as an individual imaging marker for incipient apraxia.
